# Shenkang injection for the treatment of acute kidney injury: a systematic review and meta-analysis

**DOI:** 10.1080/0886022X.2024.2338566

**Published:** 2024-04-24

**Authors:** Shengchun Liao, Yurou Chen, Shuting Wang, Chen Wang, Chaoyang Ye

**Affiliations:** a Department of Nephrology, Shuguang Hospital Affiliated to Shanghai University of Traditional Chinese Medicine, Shanghai, China; b Institute of Traditional Chinese Medicine Kidney Disease, Shanghai University of Traditional Chinese Medicine, Shanghai, China

**Keywords:** Acute kidney injury, Shenkang injection, traditional Chinese medicine, systematic review, meta-analysis

## Abstract

**Objective:**

Shenkang injection (SKI) has been widely used in China for many years for the treatment of kidney disease. The objective of this systematic review was to assess the efficacy of Shenkang injection for the treatment of acute kidney injury (AKI).

**Methods:**

A search was conducted across seven databases, encompassing data from the inception of each database through October 8^th^, 2023. Randomized controlled trials comparing SKI-treated AKI patients with control subjects were extracted. The main outcome measure was serum creatinine (SCr) levels. Secondary outcomes included blood urea nitrogen (BUN), serum cystatin C (CysC), 24-h urine protein (24 h-Upro) levels, APACHE II score and adverse reactions.

**Results:**

This meta-analysis included eleven studies, and the analysis indicated that, compared with the control group, SKI significantly decreased SCr [WMD = −23.31, 95% CI (-28.06, −18.57); *p* < 0.001]; BUN [WMD = −2.07, 95% CI (-2.56, −1.57); *p* < 0.001]; CysC [WMD = −0.55, 95% CI (-0.78, −0.32), *p* < 0.001]; 24-h urine protein [WMD = −0.43, 95% CI (-0.53, −0.34), *p* < 0.001]; and the APACHE II score [WMD = −3.07, 95% CI (-3.67, −2.48), *p* < 0.001]. There was no difference in adverse reactions between the SKI group and the control group [RR = 1.32, 95% CI (0.66, 2.63), *p* = 0.431].

**Conclusion:**

The use of SKI in AKI patients may reduce SCr, BUN, CysC, 24-h Upro levels, and APACHE II scores in AKI patients. The incidence of adverse reactions did not differ from that in the control group. Additional rigorous clinical trials will be necessary in the future to thoroughly evaluate and establish the effectiveness of SKI in the treatment of AKI.

## Introduction

Acute kidney injury (AKI) is a prevalent medical condition caused by a combination of ischemic and toxic insults. It is known for its high mortality rate, which can range from 25% to more than 50%, resulting in more than 1.7 million deaths annually. Furthermore, individuals who survive AKI may face long-term complications, such as the development of end-stage renal disease (ESRD) and chronic kidney disease (CKD) [1,29]. The glomerular filtration rate (GFR) is considered the gold standard for diagnosing acute kidney injury (AKI). However, direct measurement of the GFR is rarely conducted in clinical practice. Instead, a surrogate indicator of renal function is typically used. The serum creatinine (SCr) concentration is commonly used, but it lags behind acute changes in renal function, and estimating GFR using equations may not accurately reflect the concentration when SCr concentration changes rapidly [2]. The definition of AKI in terms of SCr, as proposed by the Kidney Disease: Improving Global Outcomes (KDIGO) guidelines, has been extensively debated. However, no serum or urine biomarkers have been found to be more reliable than SCr for indicating AKI. Therefore, the SCr definition of AKI remains the best choice for clinical research [3]. In 2012, the KDIGO published guidelines for the classification and management of AKI, defining it as a sudden decrease in renal function occurring within 7 days or less [4]. In 2017, the ADQI 16 report proposed a revised definition of AKI, aiming to establish a link between AKI, AKD, and CKD [5]. Currently, there is no effective treatment for promoting renal recovery in AKI patients. Therefore, identifying individuals at risk for AKI and recognizing AKI early are crucial, particularly in developing countries with lower economic levels and limited kidney replacement therapy (KRT) resources [6]. Although KRT is the only option for severe AKI, there is no consensus on the optimal timing of KRT, and early initiation of KRT may have negative effects on patients. The decision of when to initiate KRT should be made with careful consideration [7]. Notably, several studies have shown that alkaloids from various herbs can ameliorate AKI. Each alkaloid has different mechanisms of action and targets, suggesting that each alkaloid is promising for the prevention and treatment of AKI [8].

Previous studies have conclusively demonstrated that various traditional Chinese herbal medicines possess remarkable protective effects against AKI. These medicinal herbs exert their effects through diverse mechanisms of action, which include the inhibition of inflammation, cell apoptosis, necroptosis, ferroptosis, and suppression of oxidative stress. These findings highlight the promising potential of these herbs as innovative therapeutic agents for individuals suffering from AKI [9]. SKI is a traditional Chinese medicine injection manufactured by Xi'an Century Shengkang Pharmaceutical Company Limited in Shaanxi Province, China. It contains specific ingredients such as rhubarb, Salvia miltiorrhiza, safflower flower, and Astragalus flavone. The injection underwent phase I, II, and III clinical trials starting in 1999, and it has been widely used in clinical practice since 2004^[10]^. SKI has been shown to delay the progression of renal failure in 5/6 nephrectomized rats and improve renal failure through different mechanisms of action, including inhibition of the immune-inflammatory response, increased renal blood flow, and improvement of renal fibrosis. According to the expert consensus, SKI is recommended for the treatment of chronic kidney disease (CKD) [10]. Additionally, several clinical studies have demonstrated the apparent effects of SKI in the treatment of AKI. A network pharmacology analysis of the four components of SKI was performed to assess the effects of SKI on hypoxia/reoxygenation (H/R) human renal tubular epithelial cells and to investigate the underlying mechanisms involved. Analysis revealed that the components of SKI, which shares the endoplasmic reticulum stress (ERS) pathway with AKI, can increase HK-2 cell viability and reduce H/R-induced apoptosis [11]. Therefore, it is reasonable to assume that SKI may be effective at preventing and treating AKI. However, a systematic review of the therapeutic role of SKI in AKI is currently lacking. In this meta-analysis, our aim was to systematically evaluate the efficacy of SKI in the prevention and treatment of AKI.

## Methods

The design of this study aligns with the 2020 Preferred Reporting Items for Systematic Reviews and Meta-analysis (PRISMA) statement [12]. A revised tool to assess the risk of bias in randomized trials (RoB 2) [13] was utilized to evaluate the risk of bias. The data analysis was performed using Stata 17.0 software.

### Study selection and data extraction

We conducted a comprehensive search using the following databases: PubMed, Embase, Cochrane Library, CNKI, WanFang Data, CBM, and VIP. The search period spanned from the beginning of the available search records until October 8^th^, 2023. The search terms of all the databases used a combination of one of the terms 'Shenkang' or 'Shenkang injection' in conjunction with one of the terms 'acute renal failure', 'acute kidney injury', or 'acute renal insufficiency'.

### Protocol

The protocol was preregistered prior to conducting the review: INPLASY, no. 2023100037, DOI: 10.37766/inplasy2023.10.0037

### Inclusion criteria

The eligibility criteria for this study included a randomized controlled trial that evaluated the effectiveness of SKI compared to no SKI as a treatment for AKI. There were no restrictions on patient characteristics, such as sex, age, religion, language, or country of origin. AKI was identified using the benchmarks established by the Acute Kidney Injury Network (AKIN) in 2005 [14], which include an increase in creatinine levels of 1.5 to 2.0 times the baseline level or a creatinine increase of at least 26.4 μmol/L. These criteria also included a urine output of less than 0.5 mL/kg/hr for at least 6 h within the past 48 h. Alternatively, the criteria established by the Kidney Disease: Improving Global Outcomes (KDIGO) in 2012 [4] were used, which include an increase in serum creatinine (SCr) level of ≥0.3 mg/dl (≥26.5 μmol/l) within 48 h, an increase in SCr to ≥1.5 times the baseline level presumed to have occurred within the prior 7 days, or a urine volume of <0.5 mL/kg/h for 6 h.

In the control group, interventions included the use of conventional pharmacotherapy, as well as hormones, immunosuppressants, and renal replacement therapy. There were no limitations or constraints placed on the dosage, type, frequency, or duration of SKI treatment. Placebo trials were also included. The intervention in the experimental group consisted of a combination of SKI therapy and the other interventions used in the control group.

### Exclusion criteria

The studies mentioned below were excluded from the analysis for the following reasons: (1) Trials did not fulfill the predetermined inclusion criteria. (2) Studies that used oral or rehydration forms of herbal preparations other than SKI. (3) Patients with other types of renal disease, congenital renal anomalies, or who underwent renal surgeries. (4) Systematic reviews, conference papers, and animal experiments. (5) Duplicate publications.

### Study selection and data extraction

The primary focus of this study was the measurement of serum creatinine (SCr), with secondary measurements including blood urea nitrogen (Bun), cystatin C (CysC), 24-h urine protein (24 h-Upro), and APACHII scores. Two authors conducted independent data extraction using a predetermined search strategy. Initially, a preliminary screening by EndNote X9 software was conducted by reviewing the titles and abstracts of the retrieved articles. The purpose of this screening process was to exclude studies that did not meet the established criteria. Afterward, a thorough evaluation was conducted to determine whether the specified criteria for inclusion were met in the complete text. The two individuals in charge of this assessment compared the selected studies and resolved any discrepancies through constructive discussion or with the involvement of a third party. Subsequently, the collected data were organized into separate categories, including the author's name, year of publication, subject information, intervention details, duration, outcome assessment, and any observed adverse events. These datasets were compiled and input into spreadsheets (Table 1).

**Table 1.** Characteristics of the included studies.

Study	Sample size (T/C)	Sex(M/F)		Age(years)		Intervention		Course of treatment (d)	Outcomes	Adverse events
		T	C	T	C	T	C			
Nie 2014 [16]	21/21	13/8	14/7	69.94 ± 5.36	68.61 ± 5.19	SKI (60-100ml qd ivgtt) + control	Symptomatic rehydration therapy + mechanical ventilation	7	SCr, CysC, APACHE IIscore	Not occurred
Song 2014 [17]	20/20	14/6	13/7	64.52 ± 7.36	67.24 ± 4.25	SKI (100 ml qd ivgtt) + control	Symptomatic rehydration therapy + hemodialysis (if necessary)	7	SCr, APACHE II score, MODS score	Not mentioned
Shi 2016 [18]	33/33	17/16	18/15	58.9 ± 4.5	58.6 ± 3.9	SKI (60-100ml qd ivgtt) + control	Symptomatic rehydration therapy + ventilator therapy (if necessary)	7	SCr, BUN, CysC, 24h-Upro, β2-MG, APACHE II,24h-UV	T: 2 epigastric discomfort; 2 nausea and vomiting C: 1 epigastric discomfort; 2 nausea and vomiting
Jia 2017 [19]	38/38	21/17	22/16	27 people ≤ 40 and 11 people > 40	29 people ≤ 40 and 9 people > 40	SKI (100 ml qd ivgtt) + CDC 1# tid po + control	CDC 1# tid po + Symptomatic rehydration therapy + hormones + Immunosuppressants (if necessary)	14	SCr, Alb, BUN, 24h-Upro, NAG, KIM-1,D, V_max_, RI, PT, APTT, TT,FIB, PAI-1, t-PA, vWF, D-D	Not mentioned
Liu 2017 [20]	40/40	24/16	25/15	45.3 ± 3.8	44.9 ± 4.6	SKI (80 ml qd ivgtt) + Simvastatin (20 mg qn po) + control	Simvastatin (20 mg qn po) + Symptomatic rehydration therapy + hemodialysis (if necessary)	21	SCr, BUN, SOD, MDA	Not mentioned
Wang 2017 [21]	30/30	18/12	16/14	72.3 ± 3.2	71.5 ± 3.4	SKI (60-100ml qd ivgtt) + control	Symptomatic rehydration therapy + ventilator therapy (if necessary)	7	SCr, CysC, NGAL, hs-CRP, IL-6, APACHE-IIscore, SOFA score	Not occurred
Gao 2020 [22]	49/49	28/21	27/22	68.23 ± 4.93	67.83 ± 5.02	SKI (40-60ml qd ivgtt) + control	Symptomatic rehydration therapy + hemodialysis (if necessary)	7	SCr, CysC, APACHE IIscore	Not occurred
Li 2020 [23]	25/25	16/9	18/7	31.95 ± 5.07	32.68 ± 5.21	SKI (100 ml qd ivgtt) + control	Symptomatic rehydration therapy	28	SCr, Cys C, β2-MG, 24H-UV	Not mentioned
Wang 2020 [24]	45/45	22/23	25/20	45.8 ± 2.9	45.1 ± 2.2	SKI (100 ml qd ivgtt) + CDC 1# tid po + control	CDC 1# tid po + Symptomatic rehydration therapy + hormones + immunosuppressant	14	SCr, Alb, BUN, 24h-Upro, NAG, KIM-1	Not mentioned
Zhang 2020 [25]	48/48	34/14	31/17	56.94 ± 6.08	56.75 ± 6.23	SKI (100 ml qd ivgtt) + control	Symptomatic rehydration therapy + hemodialysis	7	24h-Upro, SCr, BUN, GFR, KIM-1, NGAL, IL-6, IL-8	Not mentioned
Chen 2021 [26]	67/67	27/40	29/38	41.46 ± 5.18	41.15 ± 4.27	SKI (100 ml qd ivgtt) + CDC 0.5 g tid po + control	CDC 0.5 g tid po + Symptomatic rehydration therapy	14	SCr, Alb, BUN, PT, APTT, TT, FIB, IL-6, TNF-α, CRP	T: 3 infected; 4 nausea and vomiting; 2 Gastrointestinal Bleeding; 2 Retention of water and sodium C: 2 infected; 3 nausea and vomiting; 1 Gastrointestinal Bleeding; 2 Retention of water and sodium

*(T, treatment group; C, control group; SCr, serum creatinine; BUN, blood urea nitrogen; CysC, cystatin c; APACHE IIscore, Acute Physiology and Chronic Health Evaluation IIscore; 24h-Upro, 24-Hour Urine Protein; β2-GM, β2-microglobulin; 24h-UV, 24 h urine volume; MODS score, multiple organ dysfunction syndrome score; Alb, albumin; NAG, N-acetyl-β-D-glucosidase; KIM-1, kidney injury molecule 1; D, diameter of renal artery; RI, renal resistive index; V_max_, the maximum systolic blood flow velocity of renal artery; PT, prothrombin time; TT, thrombin time; FIB, fibrinogen; APTT, activated partial thromboplastin time; PAI-1, plasminogen activator inhibitor-1; t-PA, tissue plasminogen activator; vWF, von Willebrand Factor; D-D, d-dimer assay; SOD, superoxide dismutase; MDA, malondialdehyde; NGAL, neutrophil gelatinase-associated lipocalin; IL-6. Interleukin-6; IL-8, interleukin-8; TNF-α, tumor necrosis factor-α; CRP, c reactive proteins; hs-CRP, hypersensitive c reactive proteins; SOFA score, sepsis-related organ failure assessment score; GFR, glomerular filtration rate; CDC, Calcium Dobesilate Capsules)*.

### Assessment of bias risk of the included studies

**Figure 1.**
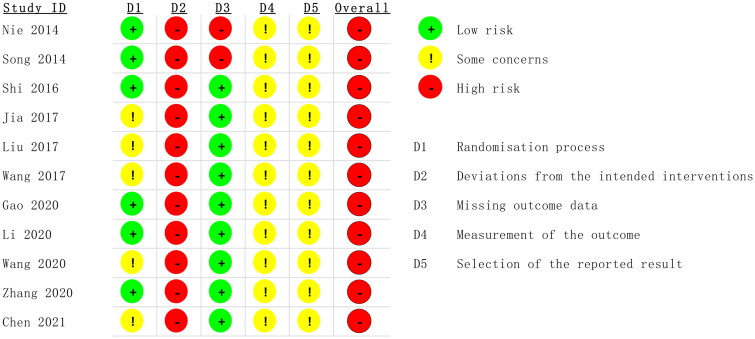
Risk bias of graph.

**Figure 2.**
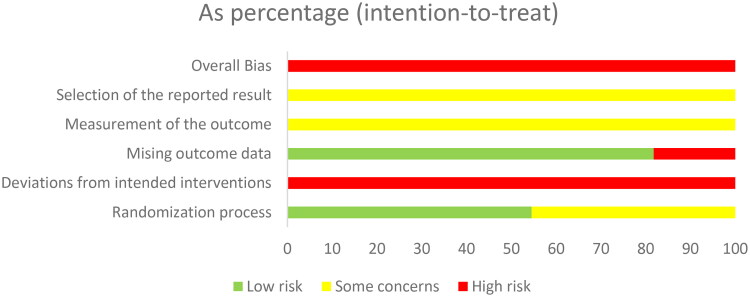
Risk bias of summary.

According to the 'A revised tool to assess risk of bias in randomized trials (RoB 2) [13]', two authors independently assessed the risk of bias in the included studies. The risk assessment consisted of five steps: (1) randomization, (2) deviation from the intended interventions, (3) missing outcome data, (4) measurement of the outcome, and (5) selection of the reported result. These items were evaluated as having a 'high risk of bias', 'low risk of bias' or 'some concerns' based on the assessment criteria (Figures 1 and 2). The quality of the evidence for each primary outcome was assessed using the Grading of Recommendations Assessment, Development, and Evaluation (GRADE) approach. Our evaluation utilized GRADEpro 3.6 software to determine the risk of bias, inconsistency, indirectness, and imprecision of the results, as well as the probability of publication bias, using a four-item scale ('Very Low,' 'Low,' 'Moderate,' or 'High'). Methodological quality was also independently evaluated by two reviewers, with a third reviewer consulted in cases of discrepancies.

### Data analysis

The analysis was conducted using Stata 17.0 software following the guidelines outlined in the Cochrane Handbook for Systematic Review of Interventions [15]. We used the relative risk (RR) with 95% confidence intervals (Cis) to present dichotomous variables. For continuous variables, we employed either the weighted mean difference (WMD) or the standardized mean difference (SMD) with 95% CI. The I^2^ statistic and χ^2^ statistic were used to evaluate heterogeneity. In cases where no significant heterogeneity was observed (I^2^ < 50% or *p* > 0.05), a fixed effects model was used. On the other hand, a random effects model was employed when heterogeneity was significant. Subgroup analyses were performed based on different interventions and baseline characteristics to explore potential sources of heterogeneity. A *p* value less than 0.05 was considered to indicate statistical significance. Additionally, publication bias was assessed using Begg's test and Egger's test. Sensitivity analyses were also conducted to examine whether the findings would have varied if the study eligibility was limited to those at low risk of bias and if alternative methods of data synthesis were used. We performed sensitivity analyses by excluding each study in turn and comparing the results of the remaining studies to those of all studies. If the studies are all consistent, the finding is shown to be robust. Furthermore, meta-regression analyses were performed to assess the associations between categorical study characteristics and the effects of the interventions.

## Results

### Search results

**Figure 3.**
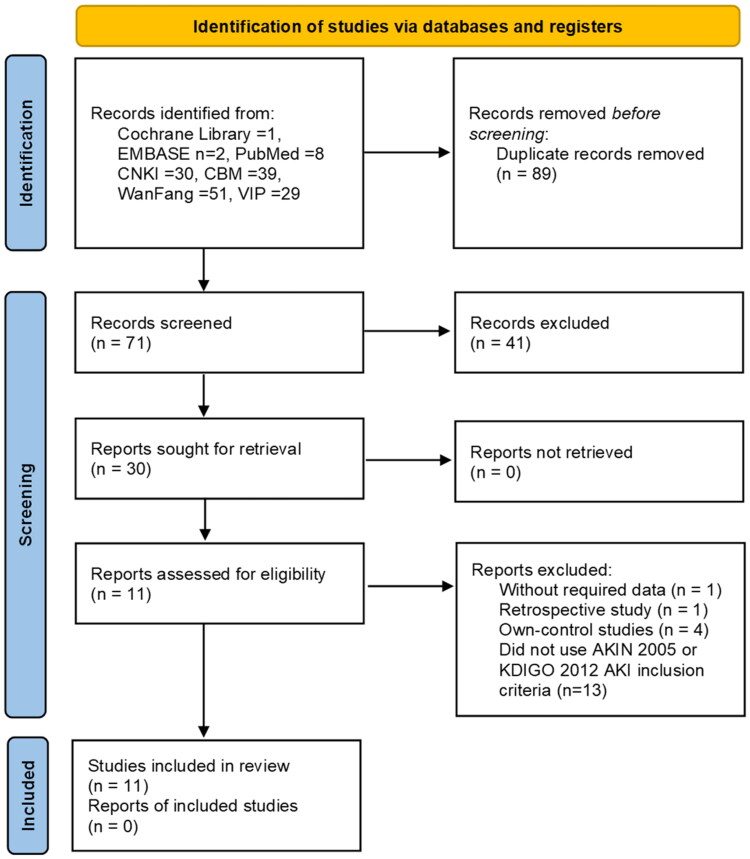
PRISMA flow diagram. SCr

A total of 160 studies were retrieved from the following databases: the Cochrane Library (*n* = 1), EMBASE (*n* = 2), PubMed (*n* = 8), CNKI (*n* = 30), CBM (*n* = 39), WanFang (*n* = 51), and VIP (*n* = 29). Of these studies, 89 were found to be duplicates. An examination of the titles and abstracts of the remaining 71 articles was also conducted, leading to the exclusion of 41 additional articles. These exclusions included 28 studies that were unrelated to the topic, 10 animal experiments, 2 conference papers, and 1 study that used other Chinese medicinal preparations. A comprehensive examination was conducted on the remaining 30 papers by thoroughly reviewing their complete text. As a result, 19 papers were excluded based on the inclusion criteria. The reasons for exclusion were as follows: 1 study did not provide the required data, 1 study was retrospective in nature, 4 studies were own-control studies, and 13 studies did not use the AKIN 2005 or KDIGO 2012 AKI inclusion criteria for income subjects. Ultimately, a total of eleven randomized controlled trials were included in the meta-analysis (Figure 3).

### Study characteristics

Eleven randomized controlled studies were included in this analysis ([16–26]]]. The study sample consisted of a total of 832 subjects, with an equal division between the experimental group (*n* = 416) and the control group (*n* = 416).

### Baselines of the included studies

A total of 832 patients were included across 11 papers, 416 in the treatment group and 416 in the control group, each with a minimum duration of treatment of 7 days and a maximum of 28 days. In the treatment group, all patients were treated with SKIs, while in the control group, all patients only received conventional therapy. One study involved a combination with simvastatin, and three involved a combination with calcium dobesilate capsules. Hemodialysis may have been part of the conventional therapy in five studies, and immunosuppressants were a possible part of conventional therapy in two studies. The data included the SCr, BUN, CysC, 24 h-Upro, and APACHE II scores and adverse reactions (Table 1).

### Quality evaluation

According to the 'A revised tool to assess risk of bias in randomized trials (RoB 2) [13]', six studies used the random number table method to generate random sequences and were considered to have a low risk of bias for random sequence generation. The remaining studies were considered with caution because they did not provide a detailed description of the random sequence generation method [19–21,26]. No article mentioned allocation concealment or blinding. Therefore, these studies were at high risk of bias [16,17]. Two studies reported dropout cases, resulting in incomplete outcome data; therefore, these studies were also assessed as having a high risk of bias. The remaining included studies did not report dropout cases or complete outcome data and were therefore assessed as having a low risk of bias. All the studies used relatively objective measures. Because it is not clear whether the studies were double-blind, there is some concern about the risk of bias. No prespecified analysis plan was found for any of the articles (Figures 1 and 2). We used the GRADE system to categorize the quality of the key outcome indicators of the 11 publications (Table 2). All outcome indicators were of low to moderate quality, with no evidence of high quality. All included metrics included studies that did not specify how blinding and allocation concealment were implemented; SCr, BUN, CysC, and APACHE II scores showed a high degree of heterogeneity, but the results were more robust; and the safety analyses, although suggestive of no adverse effects, had the potential for publication bias, suggesting that further confirmation from additional large-scale, multicenter RCTs is needed.

**Table 2.** Quality assessment.

Quality assessment							No of patients		Effect		Quality	Importance
No of studies	Design	Risk of bias	Inconsistency	Indirectness	Imprecision	Other considerations	T	C	Relative (95% CI)	Absolute		
**Serum Creatinine**												
11	randomized trials	Serious^a^	Serious^b^	no serious indirectness	no serious imprecision	None^c^	409	398		MD 23.31 lower (28.06 to 18.57 lower)	⊕⊕○○↓ LOW	Critical
**Blood Urea Nitrogen**												
6	randomized trials	Serious^a^	Serious^b^	no serious indirectness	no serious imprecision	none	271	271		MD 2.07 lower (2.56 to 1.57 lower)	⊕⊕○○↓ LOW	Critical
**Cystatin C**												
5	randomized trials	Serious^a^	Serious^b^	no serious indirectness	no serious imprecision	none	155	149		MD 0.55 lower (0.78 to 0.32 lower)	⊕⊕○○↓ LOW	Critical
**24-Hour Urine Protein**												
4	randomized trials	Serious^a^	no serious inconsistency	no serious indirectness	no serious imprecision	none	164	164		MD 0.43 lower (0.53 to 0.34 lower)	⊕⊕⊕○↓ MODERATE	Critical
**APACHE II Score**												
5	randomized trials	Serious^a^	Serious^b^	no serious indirectness	no serious imprecision	none	146	135		MD 3.07 lower (3.67 to 2.48 lower)	⊕⊕○○↓ LOW	Critical
**Adverse Reactions**												
5	randomized trials	Serious^a^	no serious inconsistency	no serious indirectness	no serious imprecision	Reporting bias [3]	15/185 (8.1%)	11/189 (5.8%)	RR 1.32 (0.66 to 2.63)	19 more per 1000 (from 20 fewer to 95 more)	⊕⊕○○↓ LOW	Important

^a^Some of the included studies did not describe how the blinding and allocation of concealment was implemented.

^b^ Although the source of heterogeneity cannot be explained, the results are robust.

^c^Egger's test indicates publication bias.

### Outcome measures

#### Serum creatinine

**Figure 4.**
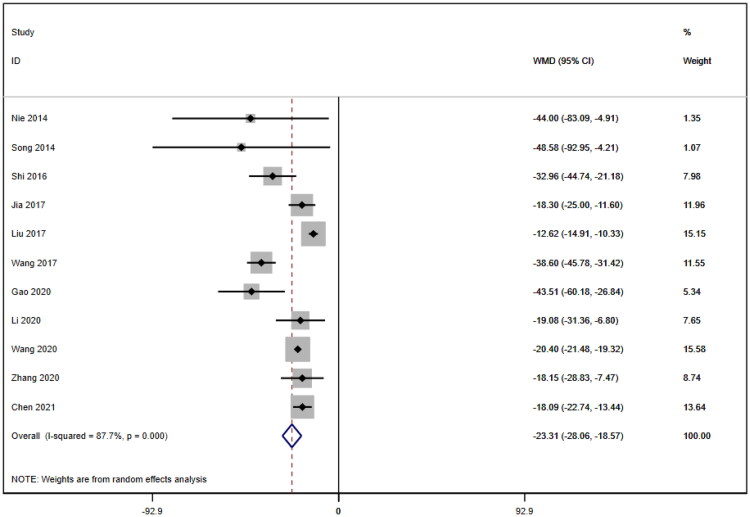
Forrest plot of SCr.

**Figure 5.**
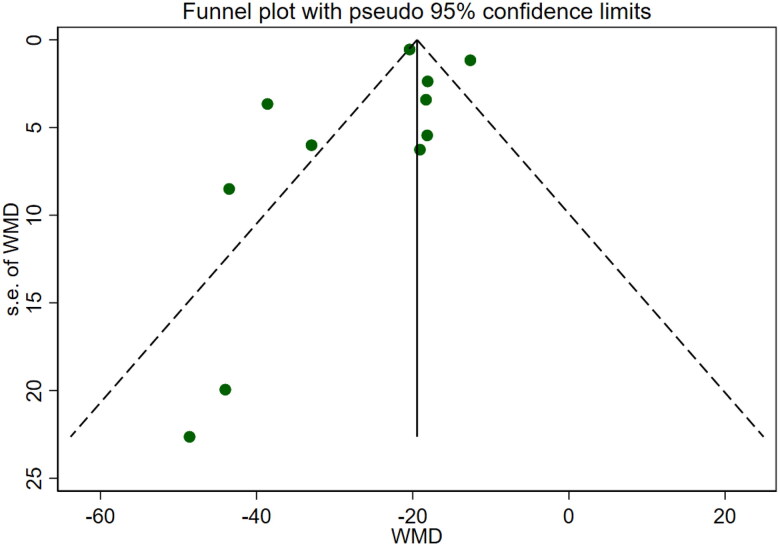
Funnel plot of SCr. Subgroup of Scr

Eleven studies [16–26] reported serum creatinine levels; these included a total of 832 patients. A heterogeneity test (I^2^=87.7%, *p* < 0.001) was also conducted, and a random effects model was utilized (Figure 4). Additionally, to ensure the thoroughness of this study, a funnel plot analysis was conducted to examine the trials included. The results of this analysis revealed the presence of potential publication bias and the inclusion of studies of inadequate quality, leading to an uneven distribution (Figure 5). Begg's test (*p* = 0.276) and Egger's test (*p* = 0.334) were also conducted, with the results indicating the absence of publication bias. Furthermore, sensitivity analyses were performed, confirming the stability of the findings. The meta-analysis demonstrated that the experimental group exhibited greater effectiveness in reducing the serum creatinine concentration than the control group [WMD = −23.31, 95% CI (−28.06, −18.57), *p* < 0.001].

##### Subgroup analysis of serum creatinine

**Figure 6.**
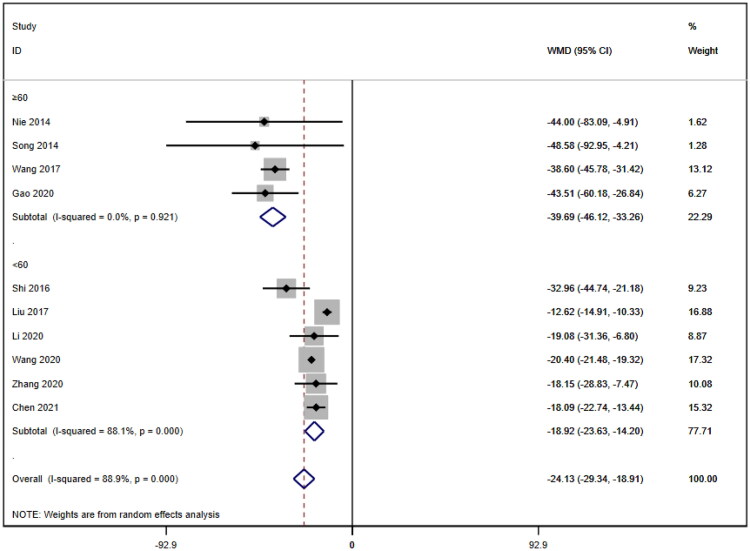
Subgroup by age of SCr.

**Figure 7.**
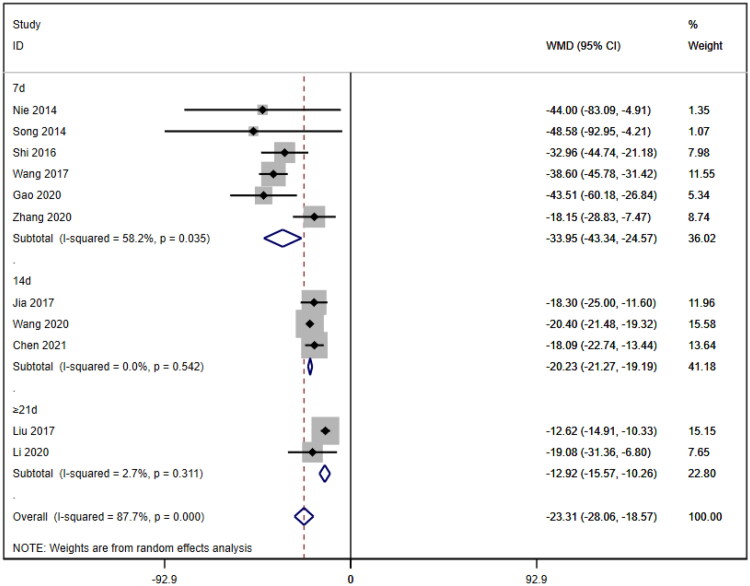
Subgroup by treatment of SCr.

**Figure 8.**
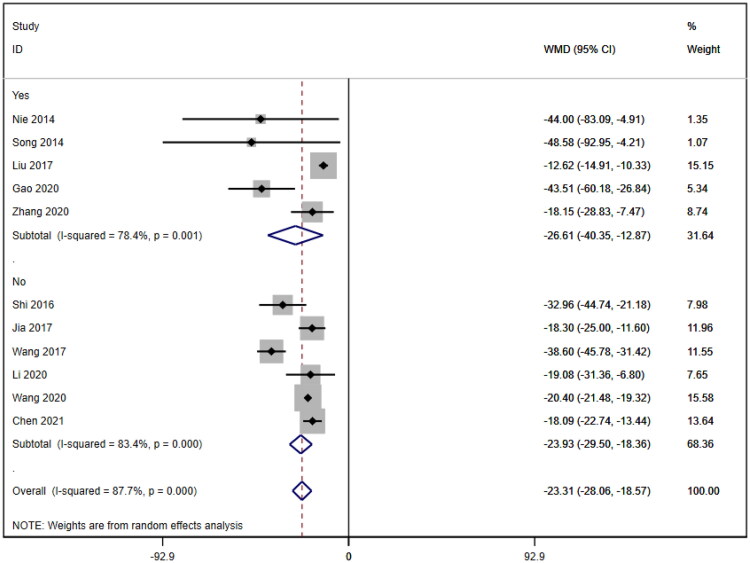
Subgroup by hemodialysis of SCr.

**Figure 9.**
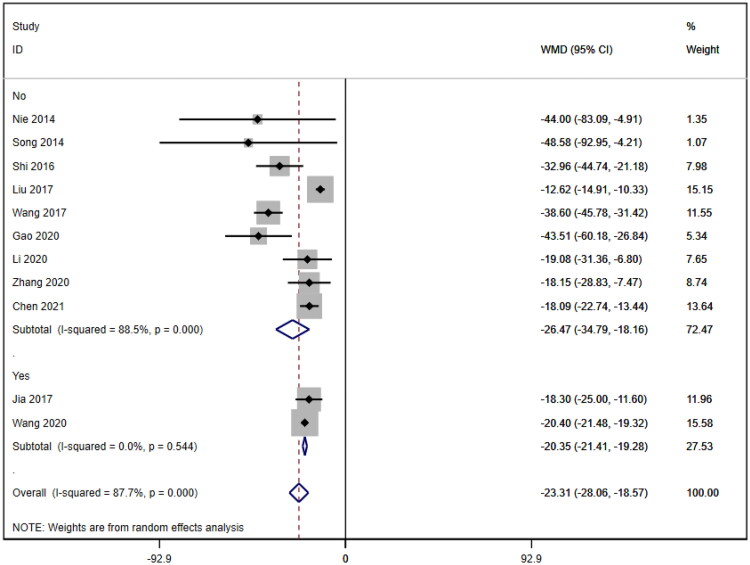
Subgroup by immunosuppressant of SCr. BUN

Subgroup analyses were also conducted on the serum creatinine concentration to account for the high level of heterogeneity. The following four subgroups were established: age (≥60 years [WMD= −39.69, 95% CI (−46.12, 95% CI, *p* < 0.001; I^2^=0.0%); <60 years [WMD= −18.92, 95% CI (−23.63, 14.20), *p* < 0.001; I^2^=88.1%]; due to inability to obtain age means, Jia (2017) [19] was excluded); treatment duration (7 days [WMD= −33.95, 95% CI (−43.34, 24.57), *p* < 0.001; I^2^=58.2%]; 14 days [WMD= −20.23, 95% CI (−21.27, 19.19), *p* < 0.001; I^2^=0.0%]; ≥21 days [WMD= −12.92, 95% CI (−15.57, 10.26), *p* < 0.001; I^2^=2.7%]); hemodialysis status (use [WMD= −26.61, 95% CI (−40.35, −12.87), *p* < 0.001; I^2^=78.4%]; no use [WMD= −23.93, 95% CI (−29.50, −18.36), *p* < 0.001; I^2^=83.4%]); and use of immunosuppressants (no use [WMD= −26.47, 95% CI (−34.79, −18.16), *p* < 0.001; I^2^=88.5%]; use [WMD= −20.35, 95% CI (−21.41, −19.28), *p* < 0.001; I^2^=0.0%]). Lower heterogeneity was not observed in any of the four subgroup analyses (Figures 6–9).

##### Meta regression of serum creatinine

Meta-regression analyses were also conducted for four subgroups: age (*p* = 0.003) (excluding [19]), treatment duration (7d *p* = 0.005; 14d *p* = 0.292), hemodialysis status (*p* = 0.899) and use of immunosuppressants (*p* = 0.420). The meta-regression results suggest that age may contribute to the heterogeneity in SCr.

### Blood urea nitrogen

**Figure 10.**
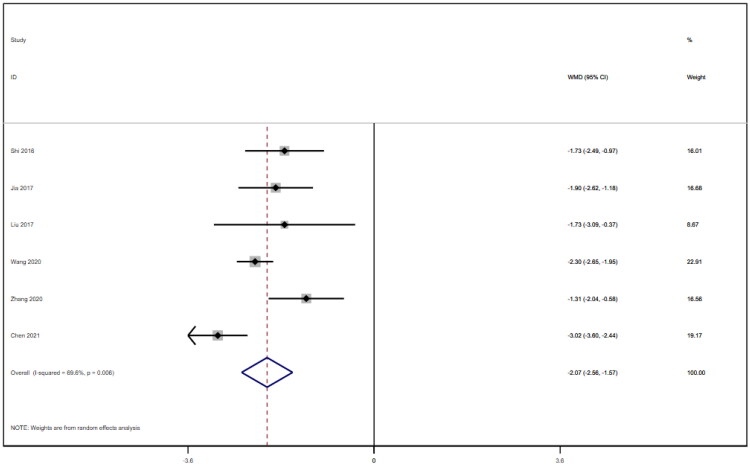
Forrest plot of BUN.

**Figure 11.**
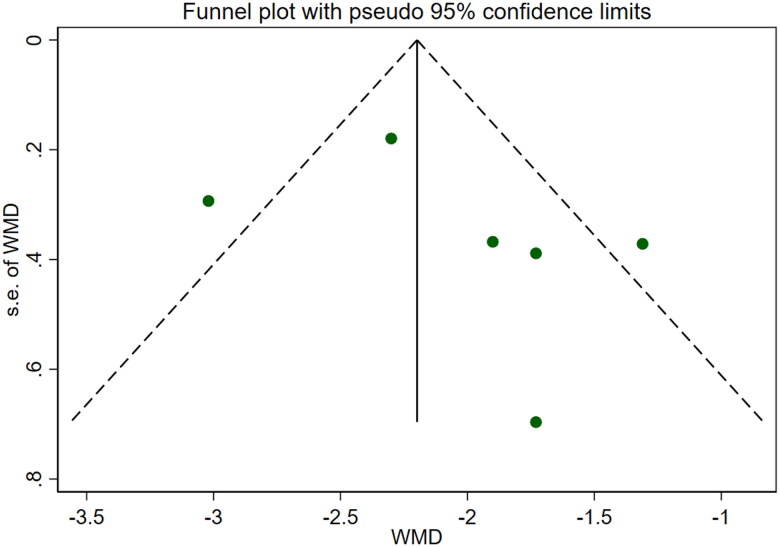
Funnel plot of BUN. CysC

Six studies [18–21,25,26] involving a total of 542 patients reported blood urea nitrogen levels. After conducting a heterogeneity test (I^2^=69.6%, *p* = 0.006), a random effects model was employed (Figure 10). To ensure the thoroughness of this research, we conducted a comprehensive analysis using a funnel plot. The analysis revealed the presence of potential publication bias and the inclusion of studies of substandard quality, resulting in an uneven distribution (Figure 11). Furthermore, Begg's test (*p* = 0.452) and Egger's test (*p* = 0.367) were performed, indicating no evidence of publication bias. Sensitivity analyses were carried out and demonstrated stable results. The meta-analysis demonstrated that the experimental treatment was significantly more effective than the control treatment in reducing blood urea nitrogen levels [WMD = −2.07, 95% CI (−2.56, −1.57), *p* < 0.001].

**Figure 12.**
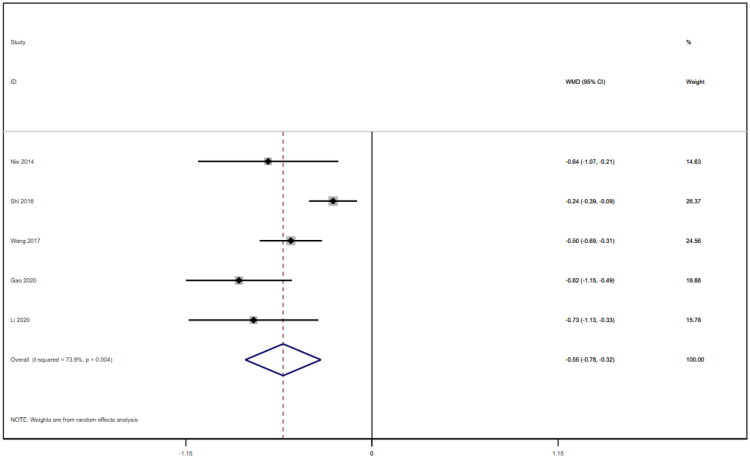
Forrest plot of CysC.

**Figure 13.**
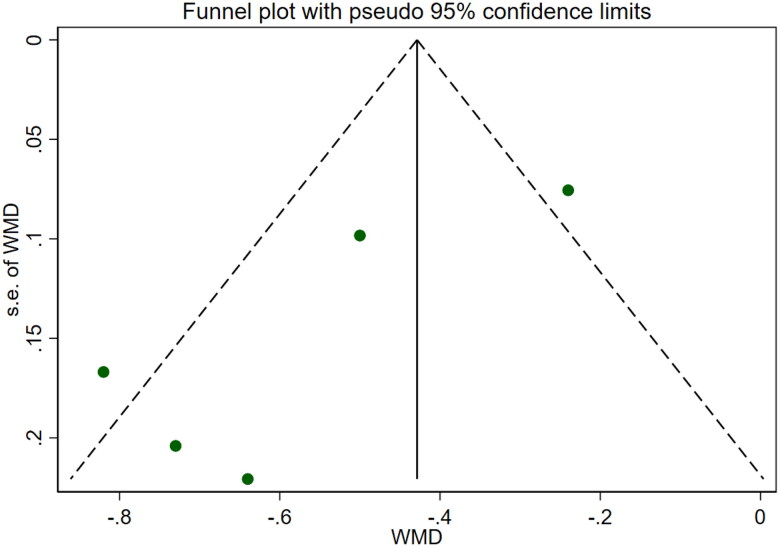
Funnel plot of CysC. 24h-Upro

### Cystatin C

Five studies [16,18,21–23] reported on cystatin c and included a total of 310 patients. After conducting a heterogeneity test (I^2^=73.9%, *p* = 0.004), we employed a random effects model (Figure 12). An analysis was conducted using the funnel plot method to evaluate the trials included in the study. The findings of this analysis revealed the potential presence of publication bias and the inclusion of studies of lower quality, resulting in an uneven distribution of data (Figure 13). To further investigate this phenomenon, Begg's test (*p* = 0.462) and Egger's test (*p* = 0.062) were conducted, and the results suggested no publication bias. Furthermore, sensitivity analyses were conducted, and the results demonstrated stability. The meta-analysis revealed that the experimental treatment was more effective at reducing cystatin c levels than was the control treatment [WMD= −0.55, 95% CI (−0.78, −0.32), *p* < 0.001].

### 24-Hour urine protein

**Figure 14.**
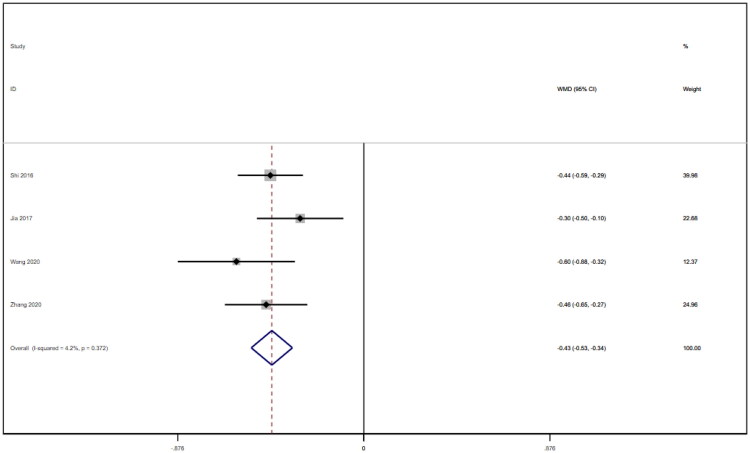
Forrest plot of 24-hour urine protein.

**Figure 15.**
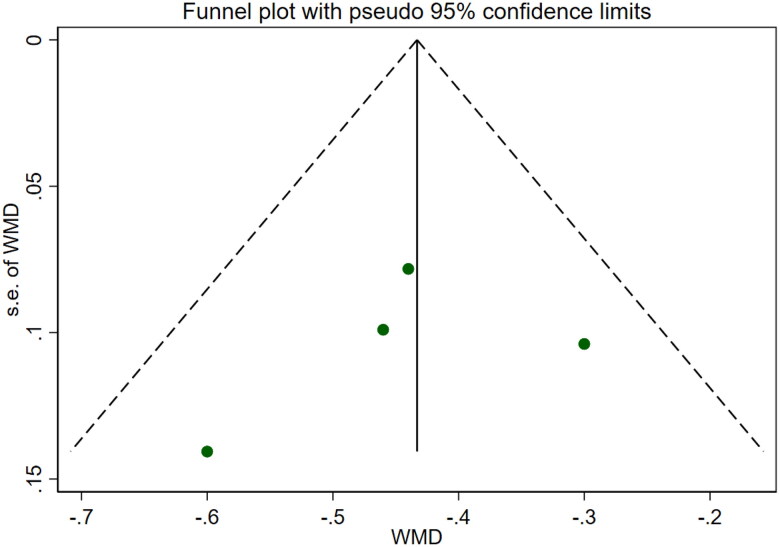
Funnel plot of 24-hour urine protein. APACHE II SCORE

Four studies [18,19,21,25] reported 24-h urine protein levels for a total of 328 patients. After conducting a heterogeneity test (I^2^=4.2%, *p* = 0.372), a fixed effects model was used (Figure 14). We used a funnel plot to examine the trials included in this study. The analysis revealed the possible presence of publication bias and the inclusion of studies of low quality, which resulted in overall asymmetry in the results (Figure 15). Begg's test (*p* = 0.734) and Egger's test (*p* = 0.620) were also conducted, and the results indicated no publication bias. Sensitivity analyses were carried out, and the results demonstrated good stability. The meta-analysis revealed that 24-h urine protein levels were more effective in the experimental group than in the control group [WMD = −0.43, 95% CI (−0.53, −0.34), *p* < 0.001].

### APACHE II score

**Figure 16.**
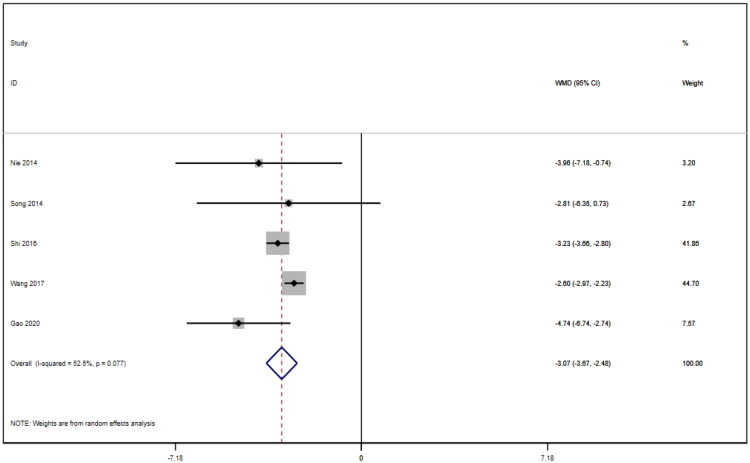
Forrest plot of APACHE II SCORE.

**Figure 17.**
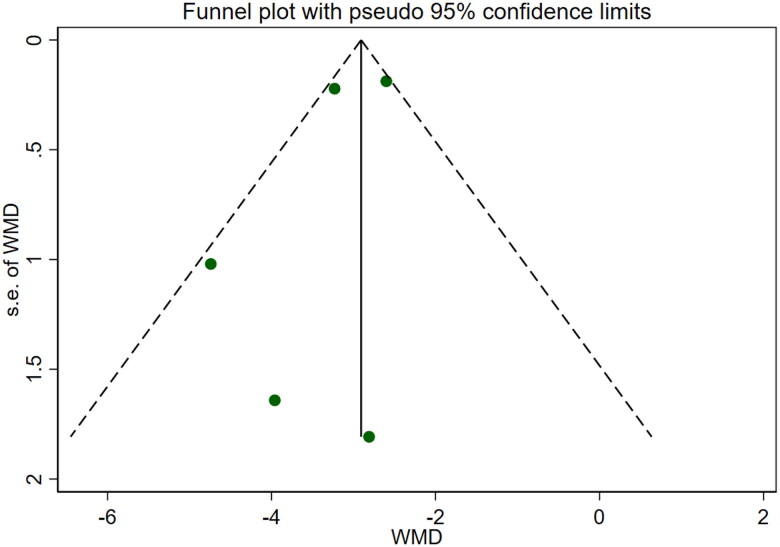
Funnel plot of APACHE II SCORE. Adverse Reactions

Five studies [16–18,21,22] reported APACHE II scores for a total of 292 patients. After conducting a heterogeneity test (I^2^=52.5%, *p* = 0.077), a random effects model was employed (Figure 16). A funnel plot was used to analyze the trials included in this study. The results indicated the presence of potential publication bias and the inclusion of studies of low quality, resulting in an observed asymmetry in the plot (Figure 17). Begg's test (*p* = 0.806) and Egger's test (*p* = 0.370) were performed, and the results indicated no publication bias. Sensitivity analyses were also conducted and showed consistent results. The meta-analysis revealed that the experimental treatment had greater effectiveness than the control treatment in terms of the APACHE II score [WMD = −3.07, 95% CI (−3.67, −2.48), *p* < 0.001].

### Adverse reactions

**Figure 18.**
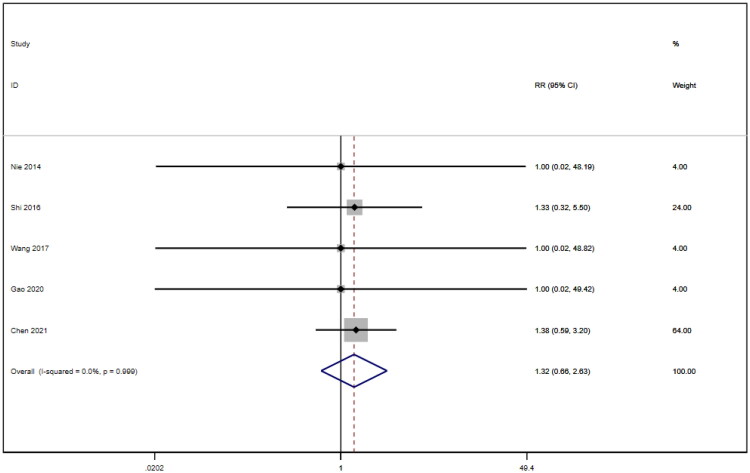
Forrest plot of adverse reactions.

**Figure 19.**
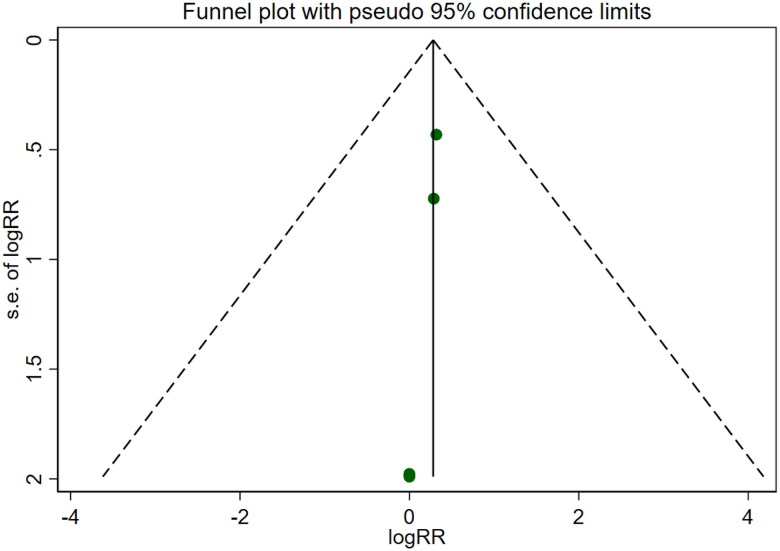
Funnel plot of adverse reactions.

Five studies [16,18,21,22,26] involving a total of 400 patients reported adverse reactions. After conducting a heterogeneity test (I^2^=0.0%, *p* = 0.999), a fixed effects model was employed (Figure 18). An analysis was conducted using a funnel plot was used to analyze the trials included in this study. The results indicated the presence of potential publication bias and the inclusion of studies of low quality, resulting in an observed asymmetry in the plot (Figure 19). Begg's test (*p* = 0.462) and Egger's test (*p* = 0.001) were performed, and the results of Begg's test showed that there was no publication bias, while Egger's test showed that there was publication bias. Sensitivity analyses were also conducted and showed consistent results. The meta-analysis revealed that the experimental treatment did not demonstrate greater effectiveness than the control treatment in terms of adverse reactions [RR = 1.32, 95% CI (0.66, 2.63), *p* = 0.431].

## Discussion

This meta-analysis included a total of eleven RCTs involving a sample of 832 patients. The main findings were as follows: (1) SCr [WMD = −23.31, 95% CI (-28.06, −18.57), *p* < 0.001]. Compared to those in the control group, patients who used SKI had significantly lower SCr levels. Due to the high degree of heterogeneity among the included studies, subgroup analyses of SCr were performed based on age, treatment duration, hemodialysis status and use of immunosuppressants. However, no source of heterogeneity could be identified. Meta-regression analysis of SCr levels was also conducted for the same subgroups, and the results suggested that age might be a potential source of heterogeneity. The impact of missing age data from the study by Jia (2017) on the source of heterogeneity remains questionable, as it was not included in the subgroup analysis or meta-regression analysis. In addition to this, we included studies with different AKI diagnostic criteria, study populations, and AKI baseline severity, which could be potential sources of heterogeneity in the findings. (2) BUN [WMD = −2.07, 95% CI (−2.56, −1.57), *p* < 0.001]; CysC [WMD = −0.55, 95% CI (− 0.78, −0.32), *p* < 0.001]; and the APACHE II score [WMD = −3.07, 95% CI (−3.67, −2.48), *p* < 0.001]. The results revealed that BUN and CysC levels and the APACHE II score were lower in patients using SKI than in those in the control group, although all showed high heterogeneity. Due to the limited number of studies included, conducting subgroup analysis and meta-regression was hindered, potentially introducing a greater degree of bias. It is crucial to consider whether the heterogeneity arises from a placebo effect or poor study quality. (3) 24-h Upro [WMD = −0.43, 95% CI (−0.53, −0.34), *p* < 0.001]: The heterogeneity of both parameters was relatively low compared to that of the other four indicators. The meta-analysis results allowed us to conclude that the 24 h-Upro of patients treated with SKI was superior to that of patients in the control group. However, the number of studies and the size of the samples involved were relatively small. However, further evidence from reliable clinical studies is needed to draw conclusive conclusions. (4) Adverse Reactions [RR = 1.32, 95% CI = 0.66, 2.63], *p* = 0.431]: Five papers specifically reported adverse reactions, and there were no serious adverse reactions. The difference between the two groups was not statistically significant. Egger's test suggested potential publication bias, but given the small number of papers involved, additional high-quality studies are needed to further confirm the safety of SKI. SKI is a Chinese herb-extracted medicine injection comprising rhubarb, Salvia miltiorrhiza, safflower flower, and Astragalus flavone and has traditionally been recommended for the treatment of CKD [10]. However, the introduction of the AKD guidelines by KDIGO as a framework bridging AKI and CKD has led many physicians to recognize that these conditions are not entirely separate diseases [5]. Consequently, attempts have been made to use SKI in AKI treatment. According to TCM theory, AKI is classified as 'uroschesis', 'edema' or 'frequent vomiting and dysuria' based on clinical symptoms. According to TCM principles, AKI is linked to the invasion of external pathogenic factors In the kidneys. The fundamental treatment for AKI involves clearing important vital organs and dissolving turbidity, activating blood circulation to dissipate blood stasis [27]. This finding aligns with the therapeutic effects of SKI. Therefore, it is reasonable to assume that SKI has a positive impact on AKI prevention and treatment, as supported by this meta-analysis.

We noted that although the results of the present meta-analysis indicated that SKIs are effective in the treatment of AKI and that the results were more robust, the results of the present meta-analysis showed smaller decreases in various renal markers and greater heterogeneity in serum markers than did previous meta-analyses of SKI for the treatment of chronic kidney disease [28]. SKI is a Chinese medicinal preparation for CKD treatment, and there is no mention of how SKI should be used for treating AKI according to the expert consensus. Based on the results of the meta-analysis, we propose the following speculations: (1) Clinicians in the clinical studies covered in this paper may have referred to the dosage of SKI in the treatment of CKD, and the duration, frequency, and dosage of medication may be insufficient for AKI treatment. Similarly, the multiple dimensions of variation that arise from this difference are likely to be a source of the higher heterogeneity in this meta-analysis. (2) Several studies have used hemodialysis in patients, which may have prematurely removed the active ingredients of SKI. (3) AKI progresses more rapidly overall than CKD, leading to a faster increase in renal markers, potentially offsetting the therapeutic effects of SKI. (4) SKI is an agent for the treatment of CKD guided by TCM theory. According to TCM theory, the treatments for CKD and AKI share many commonalities but are not identical. According to the principle of 'symptomatic treatment in acute condition, removing the primary in a chronic case', modifying the usage of SKI or even adding or subtracting the Chinese medicine components of SKI may achieve better therapeutic effects. Apart from clinical reports supporting the efficacy of SKI in AKI patients, animal experiments have demonstrated that SKI can protect AKI kidneys by restoring the oxidative balance, inhibiting the production of inflammatory factors, and up-regulating antioxidants such as SOD and GSH while down-regulating MDA levels. Pharmacological networks have indicated that PI3K/AKT, TNF, MAPK, and p53 are the main components through which SKI affects cisplatin (CDDP)-induced AKI, potentially modulating pathways associated with inflammation, oxidative stress, and apoptosis [29]. These may be potential targets for SKI efficacy in AKI. In conclusion, developing a treatment plan for SKI based on the characteristics of AKI using modern medicine and TCM may improve the effectiveness of SKI treatment.

### Limitations

Several limitations need to be considered. First, the methodological quality of the studies included in this analysis generally appeared to be low. Although all of these studies claimed to be randomized, only six reports specifically mentioned the process of sequence generation, and none of the eleven studies incorporated allocation concealment measures. Therefore, there is uncertainty regarding the effectiveness of randomization and the potential for selection bias. Second, no evidence was found in any of the studies regarding the implementation of blinding measures for participants, staff, or outcome assessments. Lack of blinding can introduce performance and detection bias. Third, all the trials included in this analysis were conducted exclusively in China, which may have created the possibility for location bias to emerge in the results. Finally, although studies with unclear diagnostic criteria for AKI were excluded, this meta-analysis used two different diagnostic criteria, namely, AKIN 2005 [16,17,22,24,26] and KDIGO 2012 [18,19–21,23,^25^]. The inclusion of different diagnostic criteria may have affected the reliability of the results. Despite these limitations, this study is the first to systematically assess the efficacy of SKI for preventing AKI and may provide useful insights for clinicians.

## Conclusions

The findings of this comprehensive analysis provide evidence supporting the potential effectiveness of SKI as a treatment for AKI. The use of SKI in patients with AKI resulted in reductions in SCr, BUN, CysC, and 24-h Upro levels and APACHE II scores. The incidence of adverse reactions did not differ from that in the control group. However, the overall methodological quality of the included research trials was relatively low. Although the study reported no difference in adverse reactions between the SKI and control groups, the safety assessment was limited. Therefore, we should approach our conclusions with caution. Additionally, further rigorous clinical trials are needed to thoroughly evaluate the efficacy of SKI in the treatment of AKI. Future studies should also focus on determining the optimal dosage and treatment duration of SKI for maximum effectiveness. Long-term follow-up studies focusing on long-term patient efficacy and safety, as well as multi-region, multi-population studies, should also be included to assess the sustained efficacy, safety and external validity of SKI for the treatment of AKI.

## Supplementary Material

Supplemental Material

## Data Availability

The study contains original contributions that are included in the article/supplementary material. For additional information, please contact the corresponding author.
